# Francisella tularensis novicida proteomic and transcriptomic data integration and annotation based on semantic web technologies

**DOI:** 10.1186/1471-2105-10-S10-S3

**Published:** 2009-10-01

**Authors:** Nadia Anwar, Ela Hunt

**Affiliations:** 1 Faculty of Biomedical and Life Sciences, University of Glasgow, Glasgow, G12 8QQ UK; 2 Department of Computer and Information Sciences, University of Strathclyde, Glasgow, G1 1XB UK; 3 Present Address: Computational Biology Center, Memorial Sloan-Kettering Cancer Center, New York, New York 10065 USA

**Keywords:** Data Integration, Database Federation, Transcriptomic Experiment, Individual Data Source, Francisella Pathogenicity Island

## Abstract

**Background:**

This paper summarises the lessons and experiences gained from a case study of the application of semantic web technologies to the integration of data from the bacterial species *Francisella tularensis novicida* (*Fn*). *Fn* data sources are disparate and heterogeneous, as multiple laboratories across the world, using multiple technologies, perform experiments to understand the mechanism of virulence. It is hard to integrate these data sources in a flexible manner that allows new experimental data to be added and compared when required.

**Results:**

Public domain data sources were combined in RDF. Using this connected graph of database cross references, we extended the annotations of an experimental data set by superimposing onto it the annotation graph. Identifiers used in the experimental data automatically resolved and the data acquired annotations in the rest of the RDF graph. This happened without the expensive manual annotation that would normally be required to produce these links. This graph of resolved identifiers was then used to combine two experimental data sets, a proteomics experiment and a transcriptomic experiment studying the mechanism of virulence through the comparison of wildtype Fn with an avirulent mutant strain.

**Conclusion:**

We produced a graph of Fn cross references which enabled the combination of two experimental datasets. Through combination of these data we are able to perform queries that compare the results of the two experiments. We found that data are easily combined in RDF and that experimental results are easily compared when the data are integrated. We conclude that semantic data integration offers a convenient, simple and flexible solution to the integration of published and unpublished experimental data.

**Electronic supplementary material:**

The online version of this article (doi:10.1186/1471-2105-10-S10-S3) contains supplementary material, which is available to authorized users.

## Background

In this paper a novel solution to flexible data integration is being examined. Semantic integration based on RDF [1] is being tested on omics data generated for the organism *Francisella tularensis* (Ft). Ft is a gram negative bacterium that causes the disease tularemia. These bacteria have the ability to cause severe disease at a low infectious dose and the potential weaponisation concerns posed by this organism have led to increased research funds to study the mechanism of virulence, which is still unknown [2]. The genomes of all four subspecies of Ft have been sequenced and compared [3]. Also, many genomic, proteomic and transcriptomic experiments have been performed on this organism. The subspecies *Francisella tularensis novicida* (Fn) strain U112 which is a less virulent subspecies of Ft, infecting only immunocompromised humans and mice, for this reason it has been well studied in the laboratory, giving rise to numerous transcriptomic and proteomic experimental data available for this subspecies.

Many experiments have focused on the Francisella pathogenicity island (FPI) and the MglA (Macrophage growth locus A) transcriptional regulator. The FPI is a 30 Kb region containing 16×19 genes whose functions remain unknown and are essential for growth within macrophage cells. Macrophages are free floating cells within the vascular system and are a part of the innate immune response. Their role is to engulf and digest pathogens in a phagolysosome, an organelle containing digestive enzymes. Normally these cells are a hostile environment for pathogens such as Francisella. However, Francisella is able to survive and replicate in macrophages by escaping the phagolysosome into the macrophage cytosol where they can replicate and ultimately escape, causing cell death. Experimental evidence shows that escape from the phagolysosome is reliant on genes encoded within the FPI [4]. In addition to the FPI, research has focused on a spontaneous mutant that is unable to disrupt the phagolysosome and replicate in the cytosol. The gene that was disrupted in this mutant was named MglA. The product of this gene regulates the transcription of genes within the FPI and approximately 90 other genes. In an attempt to understand how MglA controls the transcription of virulence factors, proteomic [5] and transcriptomic [6] experiments have been performed. This research project has aimed to understand how semantic data integration can be used effectively for Fn data. A proof of concept exercise was performed to integrate data sets from laboratories studying Fn using multiple functional genomics technologies. We focused on integration as a means to extend data annotations for experiments, using a graph of fully resolved Fn identifiers. We then used this graph of identifier cross references for the integration of proteomic and transcriptomic data.

### Motivation

Combining data from various functional genomics technologies is very difficult and is very often done by hand. The source data that are produced are stored independently of each other. Thus, gathering data on a particular pathway, organism or disease generated from various experimental technologies requires collecting and combining these data into spreadsheets, or using database software. Once these data are gathered from individual data sources, subsequent downstream analysis may be required, such as statistical tests for clustering or correlating data, or specialist algorithms that can compare the data [7]. In order for these data to be used more effectively the data at each level of analysis need to be readily accessible and easily combined. Data integration, however, is not trivial and requires resolving syntactic, structural and semantic differences across the data sources. The heterogeneity with respect to syntactic differences includes the differences in the data models such as relational databases, object stores, XML stores, flat files or spreadsheets. Structural differences lie in the data schemas that each source specifies and the query languages that they support. Semantic differences are expressed in the terminologies (vocabularies) the schemas recognise. The methodologies that are employed to overcome these problems have so far proved to be difficult to reproduce on alternative data sets and they remain to be difficult to maintain and automate. Also, since database heterogeneity is unavoidable, and a single data model (using traditional methodologies) for all biomedical data is neither probable nor possible, we require a mechanism to integrate data in an automated, scalable and flexible way.

In the majority of published studies, experimental data sources are analysed manually and data elements are manually linked to online data sources. More efficient analysis can be performed by the biologists if available online data could be easily integrated with experimental data. However, annotating every experiment, to the same extent as a genome, is very rarely performed due to time constraints. Biologists are therefore working with only part of the picture. Also, many biologists work with *unpublished data sources* and predominantly with their own experimental data. We explore the challenges biologists face while combining experimental results. Even though there is considerable database support for biological data and many systems supporting standard exchange and representation formats, not all data are available within these systems. Experimental data that are not yet published are mostly stored in their raw data formats or in spreadsheets after analysis. Databases and exchange formats have greatly facilitated data reuse and combination [8–10], however, the data available in these systems are only those data that are published. The analysis and combination of these data with experimental data is not well supported [11]. There are only very few tools and resources for combining experimental data with current knowledge and, in most cases, experimental data are annotated and combined manually, or, when the skills are available, within bespoke systems that are built to perform specific integration tasks. We propose here a semantic data integration solution that would facilitate integration of online Fn data sources with individual experimental data sets in a simple and efficient manner. We go on to show how this prototype system can be used to combine experimental data using RDF and RDF-S and we exemplify the integration and analysis of a proteomic experiment and a transcriptomic experiment. The development of the prototype allowed us to compare the two experiments and create an integrated data set that can be used for downstream statistical analysis.

In the following section we give some examples of data integration methodologies that are normally used in biological sciences. This includes a description of traditional biological integration schemes followed by an explanation of how semantic web technologies are used for data integration. Following this is a description of the construction and content of our proposed solution, *to use semantic web technologies to connect online Fn data sources through identifier mapping, then using these mapped identifiers to combine the data from two different experiments*. We also detail the conversion process for each of the data sources into RDF. We then describe the RDF-S vocabulary that was built for these data sources and how the RDF graphs combined through shared URIs. In the Utility section, we give examples of queries over the combined data and the specific queries that were performed to compare the results of the experiments. Finally, we conclude that semantic data integration offers a convenient and flexible solution to the challenges of data integration, data reuse and data accessibility in the life sciences and outline the advantages that are offered by these technologies which improve upon traditional integration methods.

### Data integration systems

The goal of a data integration system is to provide uniform access to a set of heterogeneous data sources, and to free the user from the implementation details of how data are structured at individual sources and how they are to be reconciled in order to answer queries. Data integration is most commonly achieved using one of three approaches: application integration (mediation), database federation and data warehousing [12].

Application integration involves writing special purpose software agents [13] that can query individual data sources via a single interface and then combine and return the results to the user. However, these applications can be fragile and expensive to maintain. Since integration is coded into the applications that are initially inexpensive and simple to build, these systems are notoriously fragile and susceptible to changes in the underlying systems that are being integrated. Adding new data sources often requires the application to be completely re-written. Very little integration is actually achieved through this approach. The data sources remain autonomous, queries are performed locally and the results that are gathered are combined and returned to the user. Therefore, if analysis or comparison of the data received is required this needs to be coded into the application. Portals offer another approach that is similar to application integration [14]. Usually, portals use web services to facilitate cross-database queries [15]. In these systems a query is captured by a mediating script (wrapper) which translates the query to the various data sources and returns the results to the user. Portals usually collect the data but do not integrate, rather, the data from the different sources are displayed separately within the portal interface.

The major advantage of mediation is that the application/portal delivers up-to-date data. Each source is mapped and the query mechanism is coded into a wrapper that is hidden from the user. The user accesses each source through a uniform query interface. The disadvantage to this approach is that only the queries supported by each individual system can be wrapped into the application/portal.

A more robust approach to data integration uses database federation (or mediation carried out by the database engine). Database federation describes a particular architecture where a database management system provides uniform access to a number of heterogeneous data sources. The data sources are federated, since they are linked together by the database management system. Database federation is an effective approach to the integration of heterogeneous data sources when the data can not be materialised into a data warehouse.

Data integration using a data warehouse approach, where data from the data sources are physically combined into one structure, is a very mature solution. The biggest drawback to developing a data warehouse is the scale of the resource required to integrate source data, and such data integration is usually performed piecemeal in data warehouses. Also, the integration performed by data warehouses is rarely reusable between projects. Each new project, therefore, has to perform its own data integration from scratch. Data warehouses are notoriously difficult to build, expensive to maintain and in flexible to changes in the questions that can be asked. This is largely because they require a copy to be made of data from all of the underlying data sources in a synchronised extraction, transformation and loading (ETL) process.

Data not extracted into the warehouse cannot be queried conveniently, and changing the data that are selected involves considerable redesign work. This places a large upfront design burden on the warehouse schema and the ETL process. Biological data integration requires a more flexible technology that is amenable to the ever changing landscape of biological data.

### Biological data integration

Initial solutions used to interoperate across bioinformatics databases used pre-computed cross-references or Linkouts [16]. These database cross references are used in sequence databases to link to functional annotations within other databases. For example, EMBL nucleotide database [17] cross links to protein sequence database Uniprot [18], protein function databases such as Prosite [19] and Interpro [20], protein structure databases, enzyme and pathway databases and the literature database Pubmed. These links are based on identifiers and are calculated using sequence analysis tools. Sequence databases deliver data to users via flatfile downloads and are indexed in systems such as SRS [21] and Entrez [22]. Cross references in the databases enable users to move seamlessly from one database to another. However, the databases are linked together rather than integrated.

The increased complexity of biological data and the analyses performed on these data led to the development of more complex data integration solutions. Application integration for the interoperation of data and applications became the mechanism of choice when technologies such as CORBA became popular [23]. There are also examples of federated systems, such as BioKleisli [24] which used a query language to query and manipulate data that were maintained in different formats, and DiscoveryLink from IBM [25] which provides users with a virtual database which can be accessed using SQL queries. Several data warehouse solutions have also been described [26–28]. None of these integration system can be easily extended or adapted to alternative data sets. This is mostly due to the underlying weaknesses of the technologies that were used to build the systems. Biological integration is not a solved problem. As new technologies become avaiable, the bioinformatics community exploits these with varied success. For example, semantic data integration is now in vogue [29–33] as it offers a solution to data integration that is more flexible and powerful. The advantages of semantic web technologies outlined in [29–33] make it a very attractive alternative to traditional integration.

### Semantic data integration

Rather than data integration in the traditional sense where overlapping data elements are resolved into one structure, genomic, transcriptomic and proteomic data need to be linked together using a scaffold that represents their relatedness. Semantic web technologies offer exactly this scaffold. Since genomics provides data on genes, transcriptomic experiments provide data on the transcription of genes (in particular tissues or under specific conditions), and proteomics provides identified peptides, this is not a simple case of resolving different data types and data formats. In this situation there are no common data elements between the data sets. As we deal here with data relationships which do not involve equality but different degrees of similarity or physical overlap, it is clear that traditional integration methods can not match these data in a simple manner. However, since these data are mutually related, integration can be achieved by using specific meta-data. Delivering computer understandable meta-data is the basis on which semantic web technologies were developed.

**Figure 1:**
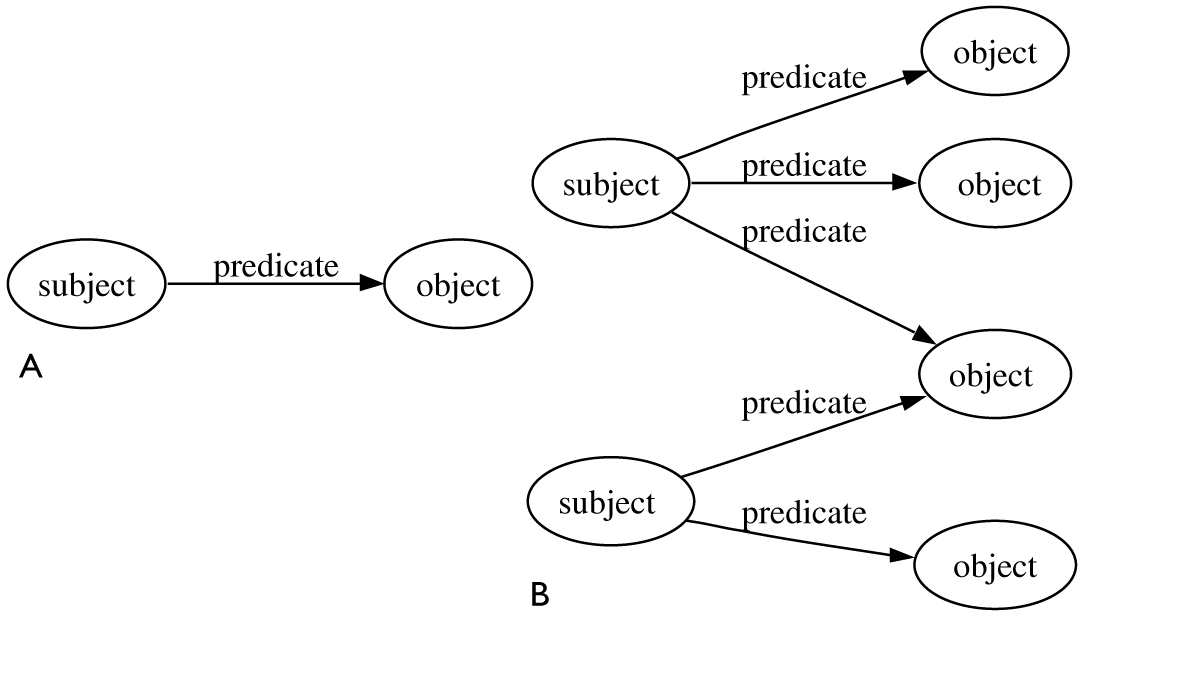
**A. RDF Triple**. B. Triples combine to form an RDF graph.

**Figure 2:**
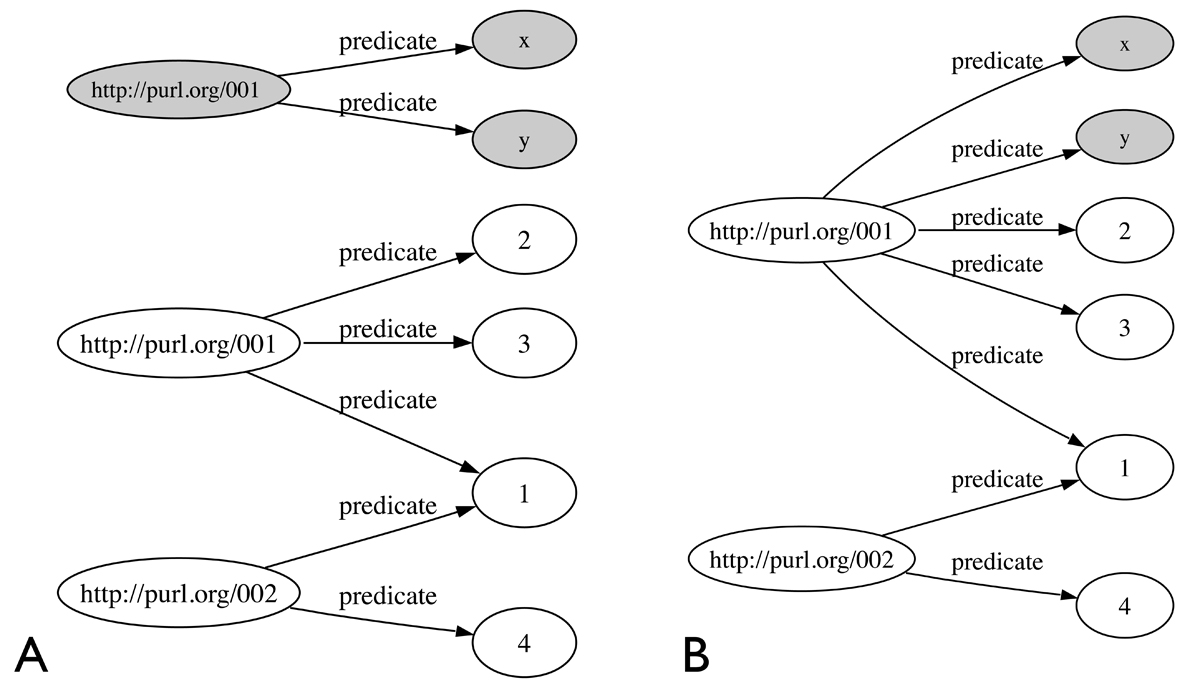
**A. Two RDF graphs with a node "http://purl.org/001"**. B. Combined graph from the graphs in Fig 2A.

A general integration model uses semantic technology layers [34] to deliver combined, machine understandable data that can be easily discovered and processed in an automated fashion. The base layers are URIs, used as unique identifiers, and RDF, the standard data representation for the data [35]. URIs (Uniform Resource Identifiers) are the base infrastructure for RDF, the Resource Description Framework. All things on the semantic web are resources, uniquely identified by URIs. For example, a URI can be given to each data element (or resource) and then meta-data (information describing the resource) is added within the RDF layer. Resources are connected to other resources via properties forming RDF triples [1]. A RDF triple consists of two resources, the subject and object, connected through a property, called a predicate (see Figure 1A). Triples can be considered to represent statements for example, "DNA is transcribed into mRNA" with DNA representing the subject, mRNA the object and "transcribed into" the predicate. RDF triples make up graphs as shown in Figure 1B. Two or more RDF graphs can be combined easily if they share URIs (see Figure 2A and Figure 2B).

The RDF-S (RDF Schema) and OWL (Web Ontology Language) [36] layers enable more heterogeneous data sources that do not share URIs to be combined. RDF-S is the vocabulary definition language for RDF. The inter-relationships between the properties and objects in RDF are defined in RDF-S. Specifically, RDF-S defines the properties and classes used in RDF graphs. Using the axioms within RDF-S, inferences about the data can be made. For example, given a set of triples with the structure "x transcribedInto y" and RDF-S statement that states that the predicate "transcribedInto" has the domain "gene" and the range "transcript", we can infer that x is a "gene" and y is a "transcript". Even though the RDF graph does not contain these triples i.e, it does not explicitly state that x is a gene and y is a transcript, this additional knowledge can be modelled in RDF-S, enabling inferences that create the triples. Such inference is useful, for example, when you want to find only genes that have some property.

**Figure 3:**
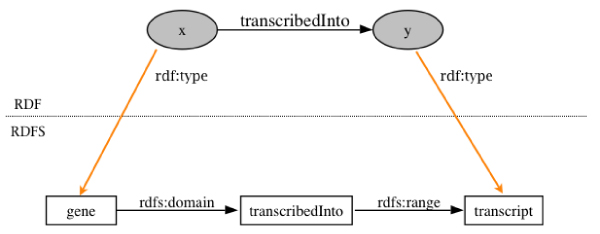
**Using RDF-S to combine data**. An example RDF triple and meta-data modelled in RDF-S. The RDF-S triples can be used, for example, to identify any nodes in the RDF graph that have RDF:type 'gene' or RDF:type 'transcript'.

Once data are combined using the standard data models RDF and RDF-S, an OWL ontology can then be used to map the relationships between the entities within the RDF and RDF-S. The rich semantics within an ontology allows the definition of detailed relationships between concepts, whereas a database schema defines only the allowed structure of a set of relations. This makes it easier to merge ontologies, or to map them to one another. Thus, further integration can be achieved between heterogeneous data sets through OWL [37]. For example, we can relate two different properties used in two RDF graphs. Given two graphs and two different predicates describing the same relationship, one graph containing triples like "x TranscribedInto 1", and the other with triples "1 transcribedFrom x", and an OWL axiom stating that "TranscribedInto" is the inverse of "transcribedFrom", when querying these two graphs, we can use either predicate to return the relevant data from both graphs (see Figure 3).

Traditional data warehouses are notoriously expensive to build and in flexible to change once deployed [38]. Data warehouses facilitate the collection, comparison and aggregation of data and the design of the warehouse is very dependent on the comparisons that are to be made, which, in turn, defines the data that are collected and how they are aggregated. New comparisons and queries can sometimes mean that new data need to be added to the warehouse, which can effect a complete re-design of the warehouse schema or other components in the warehouse structure, such as the loaders. Semantic web technologies offer a more flexible solution than data warehouses. The extraction, transformation and loading (ETL) process is greatly simplified and since there is no 'global schema' there is no expensive schema mapping stage. Therefore, adding new data sources involves only a single parsing stage (converting the data into RDF using either a Perl script or XSLT), and loading the data into the RDF store involves no complex schema mapping. Integration of updates within the RDF-S and OWL layers is potentially as simple as the addition of axioms that map the new predicates in the RDF file across the data sources, and, since this can be done incrementally, the development of data integration systems using these technologies may turn out to be more flexible and efficient. Also, with the simplified ETL process, data can be more easily refreshed and therefore there is considerably less maintenance overhead than with traditional data warehouses. For these reasons, semantic web technologies are now being widely adopted within the life sciences [30–33]. Much of the effort in this area is, however, focusing on building ontologies [39, 40] or providing semantic support within the most commonly used datasources [41–43]. There are also examples where semantic web technologies are being applied to specific diseases or organisms [33, 44]. Much of these data are already in databases or in some structured format, however, most of the data integration requirements come before data reaches databases. The effort in data integration, so far, has focused on the re-use of the data that have been analysed and published and deposited in database systems. The *integration requirements of biologists working with unpublished data are not being widely addressed by the community*.

As a proof of concept, our aims were to determine how easily data can be converted into RDF and queried. We tested semantic web technologies for data integration by first creating an RDF graph of Fn public domain data and an Fn proteomics experiment. Once this initial RDF graph was created, we then tested the flexibility of these technologies through the addition of further experimental data. We specifically focused on the integration of experimental data that are not yet published in databases, in order to provide these data into a single queryable structure that would also enable additional data to be added with ease. The two experiments that were selected were suitable for this exercise since they used two different technologies to determine the functional differences between an Fn wildtype and the Fn MgLA mutant. The integration and comparison of these two data sets provides a convenient test since these data would be difficult to integrate through traditional methodologies. Also, since the integrated data set enables the two experiments to be queried together this delivers one of only a very few comparisons of proteomic and transcriptomic data to be published so far. The integration and comparison of these data are described in the following section.

## Methods

### Data Integration using RDF and RDF-S

**Figure 4:**
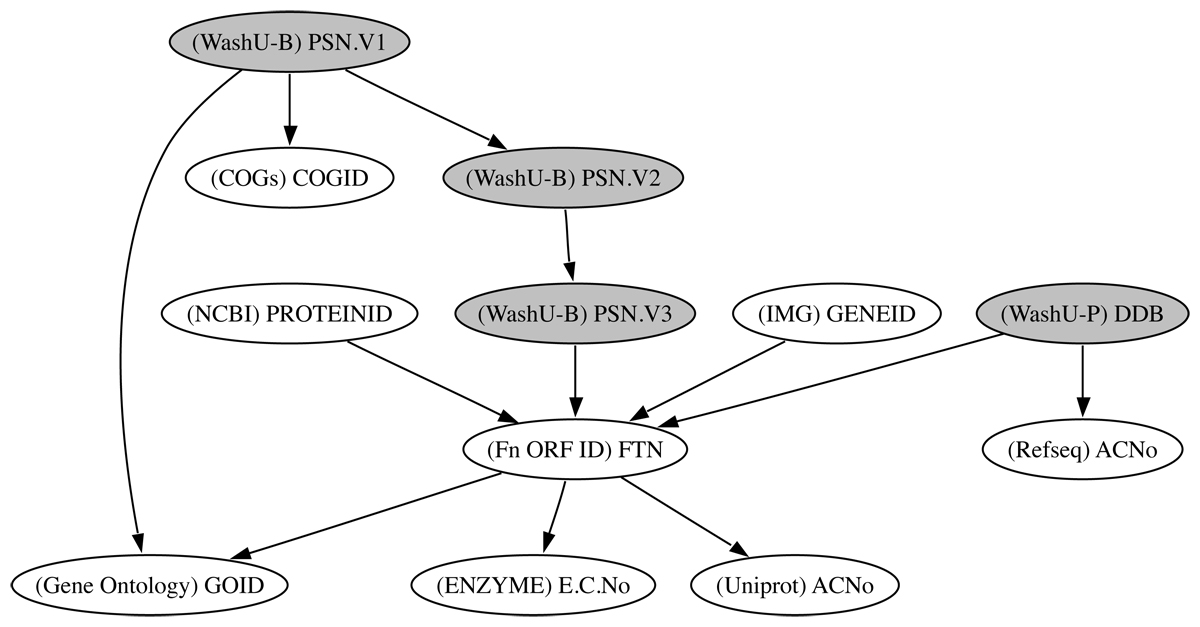
**Reconciled Identifiers in RDF**. Reconciled Identifiers in the RDF Graph, with the data source in parenthesis. This graph shows the connections that were made between the different identifiers when the data sources, given in Table 1, were combined in RDF. The shaded nodes are the identifiers used in the experimental data sets while the white nodes are the identifiers used in public domain annotations.

The main data sources used are summarised in Table 1, giving the original source information, number of triples within the RDF file and the load time into the Sesame Repository [45]. An overview of the integration architecture is shown in Figure 4.

**Table 1:** Francisella data sets combined in RDF with load times (elapsed time) into a Sesame Native repository with index structure [SPOC, POSC, POSC].

Resource	Source file name	Triples	load time
*Genome data sources and annotations*			
**Integrated Microbial Genome Data**	francisella.rdf2.nt	10,434	2.37 min
Fn genome data from The integrated microbial genomes (IMG) system			
http://genome.jgi-psf.org/mic_home.html			
**Francisella NCBI RefSeq Data**	NC_008601.nt	12,781	0.69 min
Fn annotation data from Refseq Database			
http://www.ncbi.nlm.nih.gov/entrez/viewer.fcgi?db=nuccore&id=118496615			
**KEGG genome annotation**	Pathway*.nt	113,700	3.97 min
Fn genome annotation from KEGG downloaded from PathCase			
http://nashua.case.edu/PathwaysKegg/Web/			
**Superfamily Annotation**	francisellaSUPERFAMILY.nt	16,110	0.27 min
Fn data from SUPERFAMILY Database			
http://supfam.cs.bris.ac.uk/SUPERFAMILY/cgi-bin/gen_list.cgi?genome=0b			
*Proteomics Data*			
**Proteomics Experimental Data**	Membranes.nt	416,086	10.43 min
University of Washington MglA protein abundance data sets from biological samples			
Membranes Fraction			
**Proteomics Experimental Data**	WholeCell.nt	184,221	8.36 min
University of Washington MglA protein abundance data sets from biological samples			
WholeCell Fraction			
**Proteomics Experimental Data**	Soluble.nt	580,873	4.33 min
University of Washington MglA protein abundance data sets from biological samples			
Soluble Fraction			
**Francisella (novicida U112) Proteome**		248,647	4.79 min
University of Washington Fn peptide data			
**Francisella(novicida U112) Proteome**	interact-prot.nt	20,682	0.6 min
University of Washington Fn protein identification data			
**Francisella (novicida U112) genome**	FnU112Version3.nt	56,754	1.4 min
University of Washington Fn genome data			
**Proteome Identifier mapping to Genome**	Mgla_search_db.fasta.blastp4_ypURL.nt	1,719	0.03 min
Proteomics Identifier mapping to Genome from BLAST comparison			
**GO genome annotation**	Ft_novicida_U112_go.nt	135,345	2.2 min
Fn genome annotation from Gene Ontology Database			
*Transcriptomic Data*			
**Transcriptomics Experimental Data**	GSE5468_family.soft_2.SERIES._rdf	45	0.01 min
GEO Ac GSE5468 MglA transcript abundance data set SERIES data			
**Transcriptomics Experimental Data**	GSE5468_family.soft_2.Platform._rdf	33,797	0.53
GEO Ac GSE5468 MglA transcript abundance data set PLATFORM data			
**Transcriptomics Experimental Data**	GSE5468_family.soft_2.SAMPLE._rdf	223,539	12.5 min
GEO Ac GSE5468 MglA transcript abundance data set SAMPLE data			
**Transcriptomics Experimental Data**	GSE5468_arrays_metadata.txt.nt	170	0.03 min
GEO Ac GSE5468 MglA transcript abundance data set SAMPLE metadata			
**Transcriptomics Experimental Data**	FTN2FTSA.nt	3,626	0.27 min
Blast Mapping of SchuS4 locus tag to U112 locus tag

### Francisella tularensis novicida U112 public domain data sources and annotations

The public domain data sources that were downloaded are listed in the first section of Table 1. These include the Integrated Microbial Genome Data, NCBI genome sequence and refseq data, KEGG genome data and annotation and Superfamily data. Annotations performed on the genomic data using the databases GO and COGs at the University of Washington were included within the RDF graph from the file FnU112Version3.n3. Table 2 details each public domain data source and the identifiers used within the experimental data sets from the University of Washington.

**Table 2:** Data sets and Uniform Resource Identifiers (URIs) used in the RDF graph

Resource Identifier	Source file name	No. of triples
**FTN**	Ft novicida_U112_go.nt	135,345
Fn genome annotation from Gene Ontology Database		
http://www.genome.jp/dbget-bin/www_bget?ftn:FTN_0277		
**FTN**	u112_kegg.nt	3252
Fn genome annotation from the KEGG Database		
http://www.genome.jp/dbget-bin/www_bget?ftn:FTN_0926		
**NCBI Protein ID**	NC_008601.nt	12,781
Fn annotation data from Refseq Database		
http://www.ncbi.nlm.nih.gov/entrez/viewer.fcgi?db=protein&id=118496620		
**NCBI Protein ID**	francisellaPROTEIN.fasta.nt	5,160
Fn sequence data from Refseq Database		
http://www.ncbi.nlm.nih.gov/entrez/viewer.fcgi?db=protein&id=118496625		
**NCBI Protein ID**	francisellaSUPERFAMILY.n3	16,110
Fn data from SUPERFAMILY Database		
http://www.ncbi.nlm.nih.gov/entrez/viewer.fcgi?db=protein&id=118496617		
**IMG Gene ID**	francisella.rdf2.nt	10,434
Fn genome data from The integrated microbial genomes (IMG) system		
http://img.jgi.doe.gov/cgi-bin/pub/main.cgi?section=GeneDetail&gene_oid=639753598		
**PSN.V1**	Membranes.nt/Soluble.nt/Wholecell.nt	748,157
University of Washington MglA protein abundance data sets from biological samples Membranes, Soluble and Whole cell		
https://wwamirce.gs.washington.edu/cgi-bin/fnu112/poson.cgi?poson=PSN081056		
**PSN.V1**	cogNumberURL.nt	2,54
University of Washington MglA annotation referring to COG database		
https://wwamirce.gs.washington.edu/cgi-bin/fnu112/poson.cgi?poson=PSN035866		
**PSN.V3**	FnU112Version3.nt	56,754
Fn genome data from University of Washington		
https://wwamirce.gs.washington.edu/cgi-bin/fnu112/poson.cgi?poson=PSN0088754.3		
**DDB ID**	interact-prot-peptides.nt	248,647
Fn peptide data from University of Washington		
http://regis-web.systemsbiology.net/protXML/protein_group/protein/peptide/id/ddb000010839p39		
**DDB ID**	interact-prot.nt	20,682
Fn protein identification data from University of Washington		
http://regis-web.systemsbiology.net/protXML/protein_group/protein/peptide/id/ddb000010839		
**DDB ID**	Mgla_search_db.fasta.blastp4_ypURL.nt	1,719
DDB/PSN mapping from BLAST comparison run locally		
http://regis-web.systemsbiology.net/protXML/protein_group/protein/protein_name/ddb000147854		

**Figure 5:**
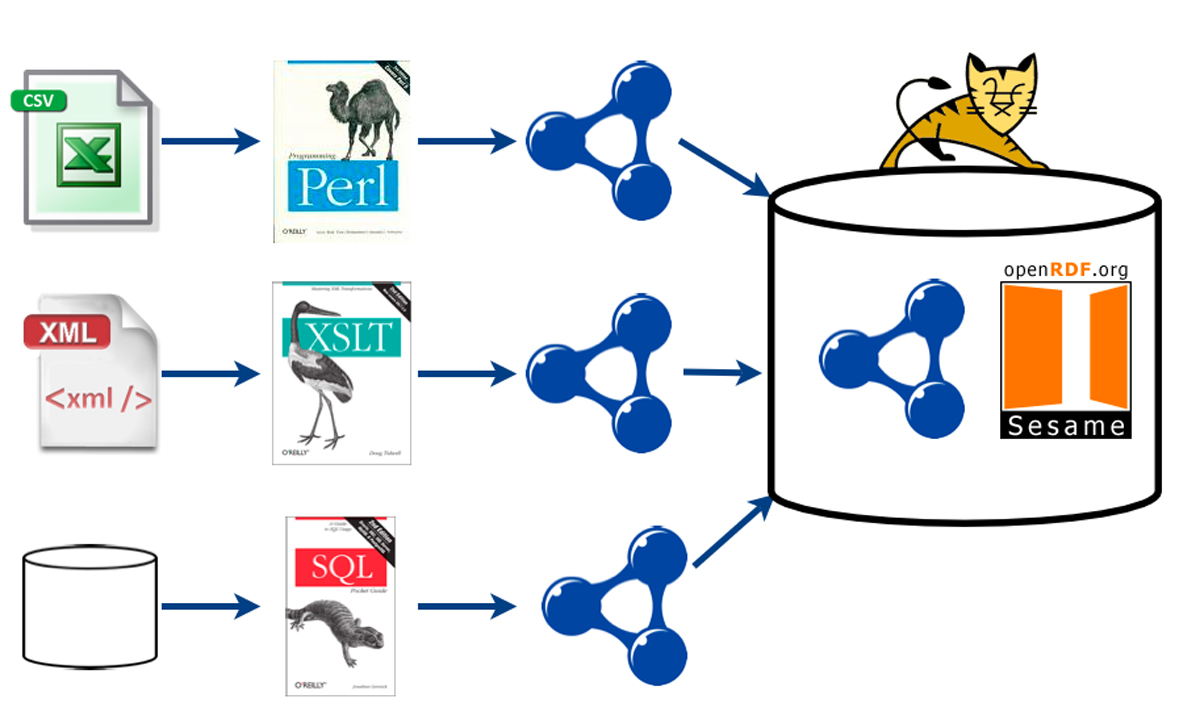
**Overview of the Integration Architecture**. Data are transformed from their native file formats, usually spreadsheets, XML or from relational tables, into RDF ntriple format. The RDF triples are then loaded into a triple store (we used the Sesame RDF store). The RDF can then be queried using the seRQL query engine or through the Tomcat HTTP interface provided by Sesame.

The combined RDF graph of Fn online data sources can be used as a source for database cross references. A graph showing how these identifiers reconcile is shown in Figure 5. The Fn genome and annotation data sources were added into the repository first, and the FTN IDs from the genome sequence, IMG [46] Gene IDs and NCBI [47] Protein IDs were connected through the CONSTRUCT statement shown in Table 3. Further data sources were subsequently added and connected to the graph (Gene Ontology data, Fn KEGG data and annotations using COGs derived at the University of Washington). These combined data enabled us to test the hypothesis that a connected graph of identifiers could increase the depth of annotation available to experimental data sets (see Utility section below). These data sets used a variety of identifiers. The UW genome data (file, FnU112Version3) used internal identifiers called POSON numbers (PSN). There were several versions of these identifiers used internally, and the proteomics experimental data generated using the MglA mutant strain (files, Membranes.nt/Soluble.nt/Wholecell.nt) used a different version of these identifiers. Data that mapped across the POSON versions were added to the graph, which enables the internal genome data and the Fn data graphs to connect. A third data set from a separate lab at the University of Washington used a third identifier, DDBs. These data were mapped to the existing identifiers through the addition of data from a BLAST [48] search against the genome data, with sequence identity set to 100%.

**Table 3:** SeRQL http://www.openrdf.org/doc/SeRQLmanual.html CONSTRUCT statement connecting the identifiers FTN IDs, IMG Gene IDs and NCBI Protein IDs. The query uses two path expressions in the FROM clause. The connection between Protein IDs and Gene IDs is made through the FTN identifier.

CONSTRUCT {proteinid} nwrce:hasGeneID {geneid} FROM
**{proteinid} G:locus_tag {ftn},**
**{geneid} G:locus_tag {ftn}**
WHERE protein LIKE "http://www.ncbi.nlm.nih.gov*"
AND geneid LIKE "http://img.jgi.doe.gov*"
USING namespace G = <http://img.jgi.doe.gov/schema#>,
nwrce = <https://wwamirce.gs.washington.edu/fnu112/schema#>

### MglA proteomics data

**Figure 6:**
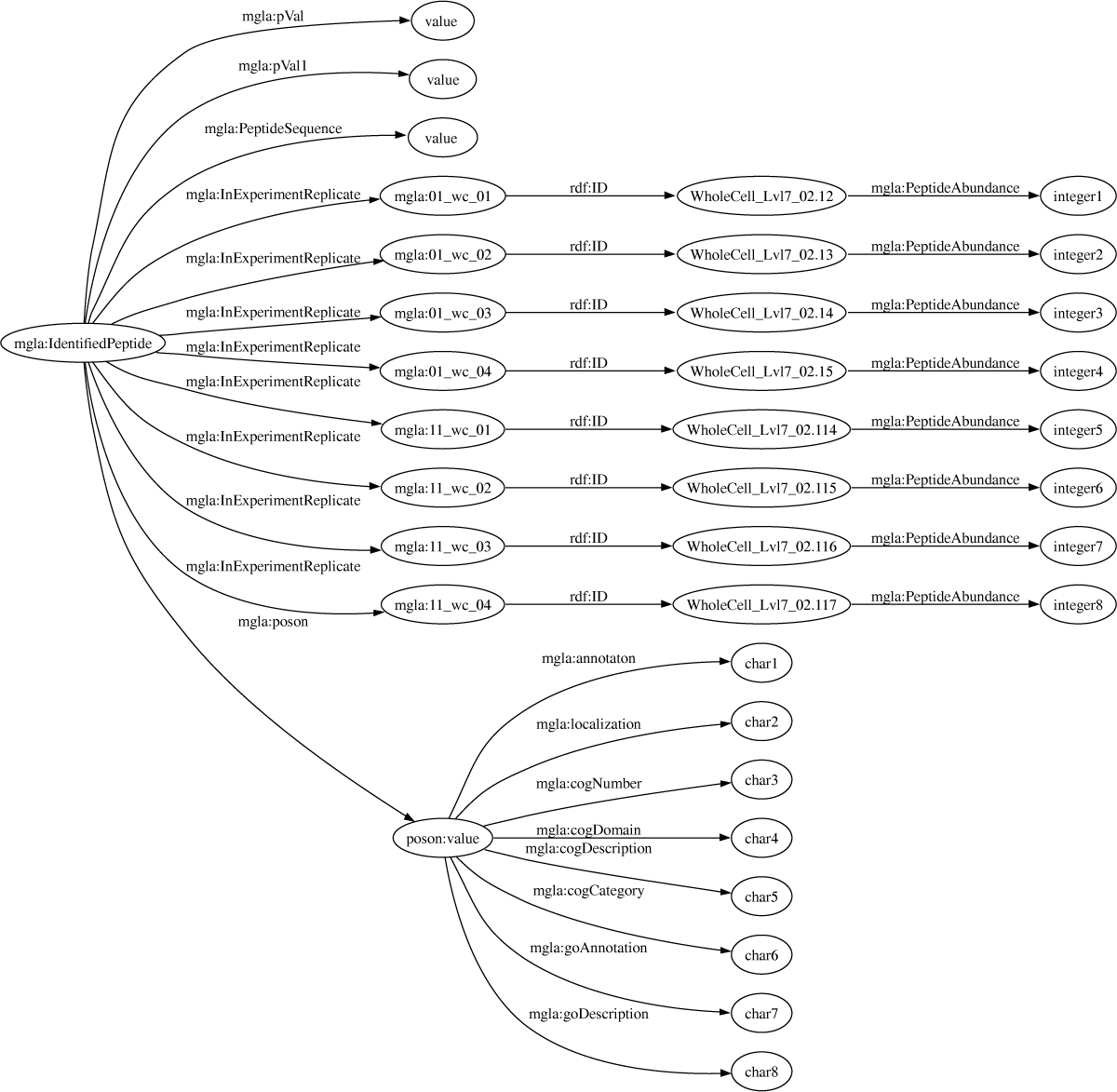
**Graph of the RDF structure for the whole cell fraction of the MglA experimental data**. The MglA experimental data were parsed from three separate spreadsheets. The RDF structure for the whole cell data are shown graphically displaying reified statements that associate peptide abundance with the specific experiment replicate in which it was measured.

The proteomics experiments [5] consisted of three spreadsheets, one for each cell fraction. The files were saved as tab delimited text and converted into the RDF model given in Figure 6, using Perl scripts (see Additional File 1). The header and one row of the spreadsheet are shown below. The first column is the identified peptide, followed by the abundance within the experimental replicates: 01_WC_1, 01_WC_2, 01_WC_3, 01_WC_4, 11_WC_1, 11_WC_2, 11_WC_3, 11_WC_4. The experimental identifiers have the structure XX_YYY_Z where XX is 01 for the wildtype and 11 for the MglA mutant, YYY represents the fraction i.e., WC (whole cell), MEM (membrane fraction) or SOL (soluble fraction) and Z represents the replicate number. This is followed by the P-Value and its complement, the PSN (poson identifier) for the protein that was identified from the peptides, and functional annotations from COG and GO.

01_WC_1 01_WC_2 01_WC_3 01_WC_4 11_WC_1 11_WC_2 11_WC_3 11_WC_4 Pval 1-Pval YYNIIYDLIDDVKK 2810 1803 3150 3593 5872 6661 4484 3746 0.021393435 0.978606565 PSN042581 protein chain initiation factor IF-2 "COG0532, InfB, Translation initiation factor 2 (IF-2; GTPase) [Translation, ribosomal structure and biogenesis]" GO0003743-translation initiation factor activity GO0006413-translational initiation Cytoplasmic

### MglA transcriptomic data from GEO accession GSE5468

**Figure 7:**
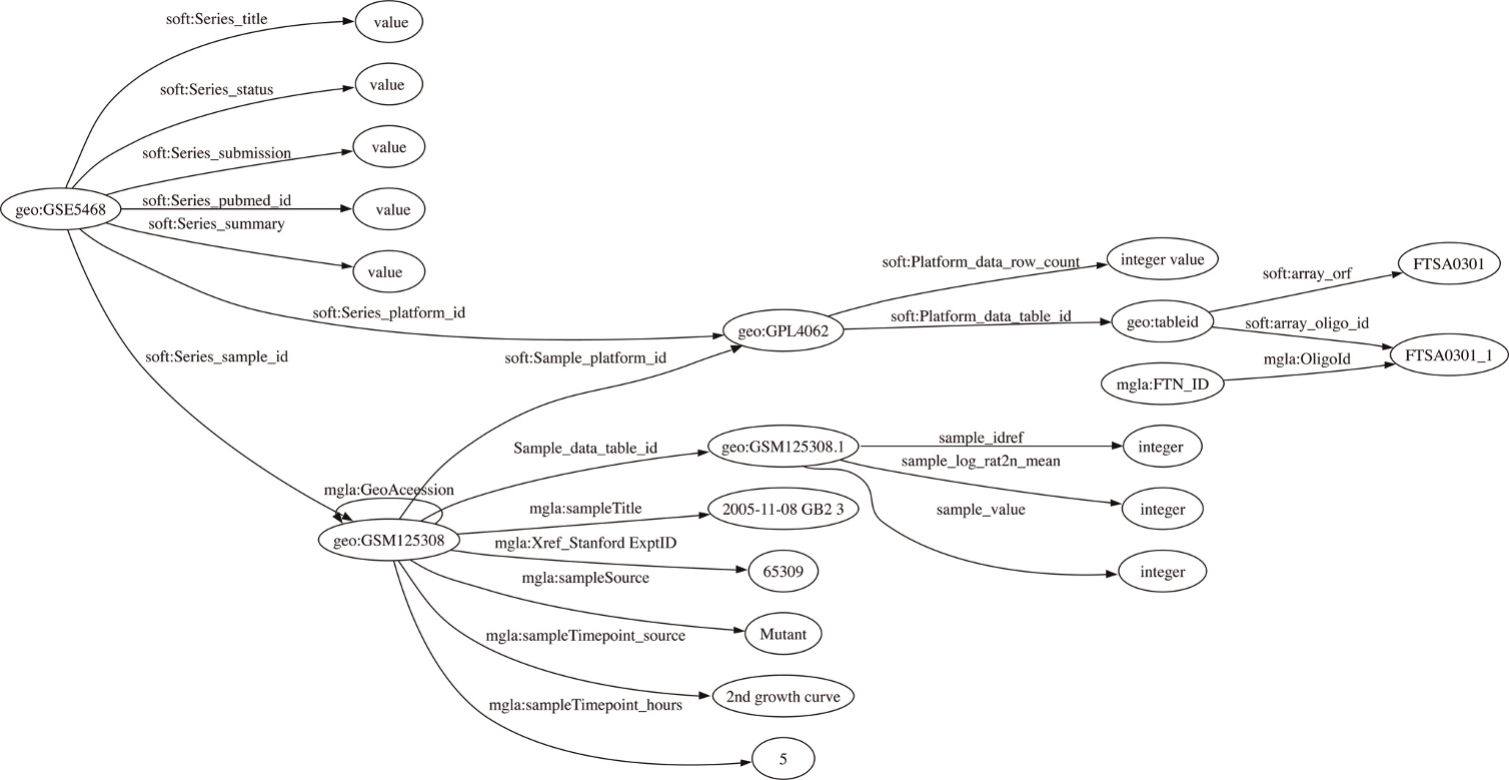
**Graph of the RDF structure for the Transcriptomics Mgla Experiment downloaded from GEO**. The GEO data were downloaded in soft format from http://www.ncbi.nlm.nih.gov/geo/query/acc.cgi?acc=GSE5468. These files were parsed into the RDF statements shown here.

This transcriptomic experiment was described in [6] and published in GEO under the accession number GSE5468 http://www.ncbi.nlm.nih.gov/geo/query/acc.cgi?acc=GSE5468. GEO data were downloaded as "SOFT formatted family files". "GSE5468_family.soft" file is split into three sections: Series, Platform and Sample. Series is an overview of the experiment and links to the sample records through the sample IDs GSMXXXX. Each Sample record associated with the experiment describes the conditions for the sample and gives the measurement made from the array. The array is described in the Platform section. This was a dual channel array: the Cy3 channel were the RNAs extracted from reference samples (see methods in [6]) and the Cy5 channel were from 10 time points taken during the 1st growth curve in hours at (0.5, 2, 2.5, 3, 3.5, 4, 4.5, 5, 7.5, 9) and seven samples during the 2nd growth curve at time in hours (1, 3, 5, 6, 7, 8, 10). The ORFs that are represented on the array are referred to using identifiers of the form "FTSAXXXX" representing locus tags from the SCHUS4 genome sequence. To link the FTSA locus tags with the locus tags of the novicida subspecies (locus tags FTN_XXXX), the locus tags were mapped using BLAST. The data from the GEO file and the mappings were extracted and transformed into the RDF structure shown in Figure 7 using a Perl script (see Additional file 2). Each sample file formed a separate RDF graph.

### RDF Schema

The data sets described above were converted into RDF using predicates derived from the data download. For example, the genome data from Integrated Microbial Genome Database uses the predicates (properties) http://img.jgi.doe.gov/schema#genomic_location_start, http://img.jgi.doe.gov/schema#genomic_location_end, http://img.jgi.doe.gov/schema#genomic_location_strand, http://img.jgi.doe.gov/schema#locus_tag and http://www.w3.org/2000/01/rdf-schema#comment, to capture the description. These predicates reflected exactly the column names used in the original tab-delimited file download with the namespace http://img.jgi.doe.gov/schema#, added to make a valid URI. The transcriptomics data used predicates from SOFT format [49]. The experimental proteomics data from the University of Washington were converted into RDF using predicates acquired from the protXML DTD http://sashimi.sourceforge.net/schema_revision/protXML/protXML_v3_xsd. As the MglA proteomics experiment was not available in a standard format, we created predicates for this data set and defined them within and RDF Schema (RDF-S). The schema is a simple class hierarchy, including properties with the relevant class domains and ranges (Table 4).

**Table 4:** RDF-S Properties with their Domain and Range values. Namespace mgla = http://www.francisella.org/novicida/schema/fnu112/experiments/mgla/

Domain	Property	Range
mgla:IdentifedPeptide	mgla:poson	mgla:IdentifedPoson
mgla:IdentifedPeptide	mgla:PeptideSequence	xsd:string
mgla:IdentifedPoson	mgla:IdentifiedFrom	mgla:IdentifedPeptide
mgla:IdentifedPeptide	mgla:IdentifiedIn	mgla:ExperimentReplicate
mgla:wildtype	mgla:ExtractedFrom	mgla:ExperimentReplicate
mgla:mutant	mgla:ExtractedFrom	mgla:ExperimentReplicate
-	mgla:PeptideAbundance	xsd:integer
mgla:IdentifedPeptide	mgla:InExperimentReplicate	mgla:ExperimentReplicate
mgla:IdentifiedPoson	mgla:annotaton	
mgla:IdentifiedPoson	mgla:localization	
mgla:IdentifiedPoson	mgla:cogNumber	
mgla:IdentifiedPoson	mgla:cogDomain	
mgla:IdentifiedPoson	mgla:cogDescription	
mgla:IdentifiedPoson	mgla:cogCategory	
mgla:IdentifiedPoson	mgla:goMolecularFunction	
mgla:IdentifiedPoson	mgla:goBiologicalProcess	
mgla:IdentifiedPoson	mgla:goAnnotation	
mgla:IdentifiedPoson	mgla:goDescription	

### Data load

The RDF Schema and the RDF Data were loaded into a Sesame [50]Native store with RDF Schema inferencing, the index structure used was [SPOC, POSC, POSC] (see Sesame user documentation, chapter 8 [45]. Data load times (elapsed time) for RDF files are shown in Table 1. Queries using the Sesame SeRQL query engine (see Chapter 9 in [45]) are described in the following section.

## Results

### Extending annotation via linked identifiers

#### Data integration increases the depth of bioinformatics annotation and reduces the effort required to manually annotate data in individual data sources

The depth of annotation available to the experimental data sets was increased through data integration based on database cross references. The RDF graph of the experimental data can be queried via the interposed layer of GO, KEGG and Superfamily descriptions, even though these data were not manually matched to these databases and provided with explicit annotations. These annotations are available by integrating data sets that have been manually annotated previously to at least one data source in the RDF graph. This form of data integration increases the amount of information available to biologists who now do not have to manually create each individual database cross reference. Sample SeRQL queries that show how the MglA experimental data are linked to annotations are shown in Table 5 for KEGG and Table 6 SUPERFAMILY data sets.

**Table 5:** SeRQL select query identifies PSNs and their E.C. numbers, where MglA peptide abundance is greater than 2000. KEGG database annotations are linked to the PSN identifiers in the MglA data through the FTN identifiers. The path expression used is displayed in bold and shown in Figure 7. Peptide abundance data is connected through the PSN identifiers.

SELECT psn, ec
FROM
**{ftn} rdfs:seeAlso {ec},**
**{psn} rdfs:seeAlso {ftn},**
**{analysis} wu:poson {psn** *}* **,**
**{analysis} mgla:experiment {exp},**
**{exp} mgla:abundance {abundance}**
WHERE abundance >2000
USING NAMESPACE
mgla = <https://wwamirce.gs.washington.edu/fnu112/experiments/mgla/schema#>,
wu = <https://wwamirce.gs.washington.edu/fnu112/schema#>

**Table 6:** Superfamily Annotation Query. SeRQL select query identifies PSNs, NCBi Protein identifiers and Superfamily annotations from SUPERFAMILY database, where MglA peptide abundance is greater than 2000. SUPERFAMILY database annotations are linked to the PSN identifiers in the MglA data through the FTN identifiers which are linked to the NCBI Protein identifiers. The path expression is displayed in bold and shown graphically in Figure 8.

SELECT psn, pid, family
FROM
**{psn} rdfs:seeAlso {ftn},**
**{pid} gen:locus tag {ftn},**
**{pid} prot:Protein Family {family},**
**{analysis} wu:poson {psn},**
**{analysis} mgla:experiment {exp},**
**{exp} mgla:abundance {abundance}**
WHERE abundance > 2000
AND family LIKE "http://supfam.org/SUPERFAMILY/cgi-bin/model.cgi?model=*"
USING NAMESPACE gen = <http://img.jgi.doe.gov/schema#>,
prot = <http://purl.uniprot.org/core/>,
mgla = <https://wwamirce.gs.washington.edu/fnu112/experiments/mgla/schema#>,
wu = <https://wwamirce.gs.washington.edu/fnu112/schema#>

#### Querying MglA proteomics data through KEGG

**Figure 8:**
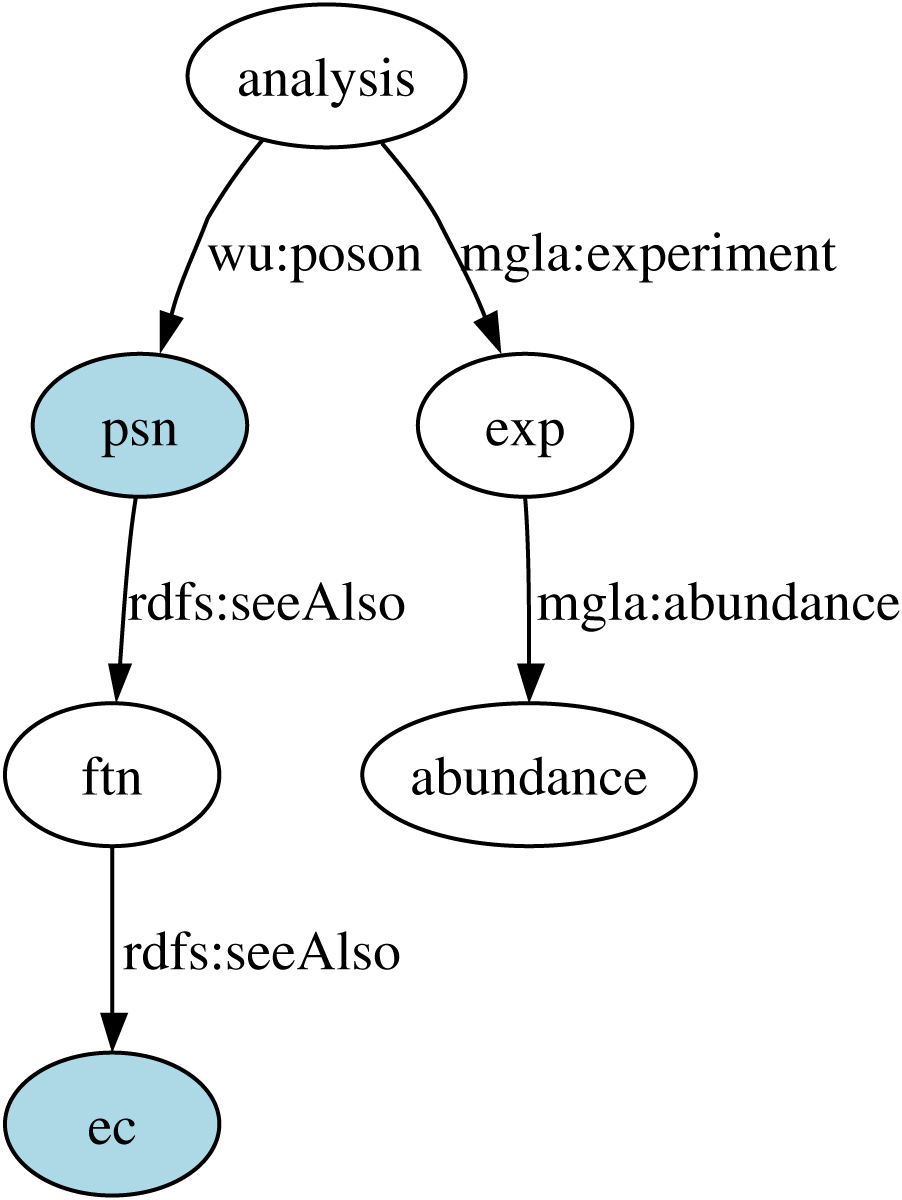
**Path expression used in the query given in Table 5**.

The query shown in Table 5 and depicted in Figure 8 gives the PSN identifiers and their E.C. numbers from the KEGG database for PSNs whose abundance in the MglA experiment was above 2000. The MglA data was not annotated using KEGG data. These links are available through the identifier cross references established in the RDF graph.

#### Querying MglA proteomics data through superfamily

**Figure 9:**
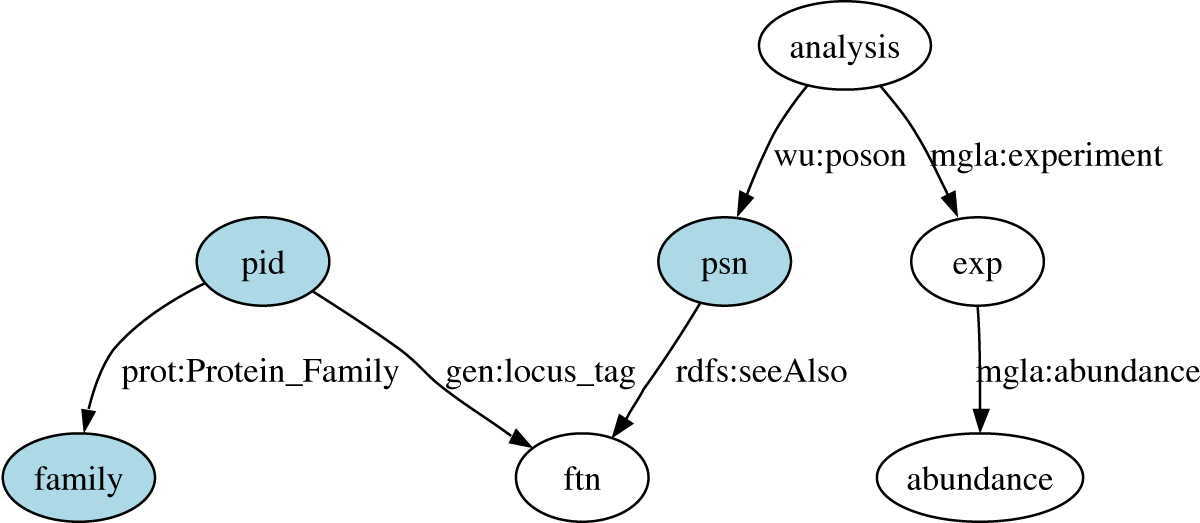
**Path expression used in the query given in Table 6**.

The query in Table 6 shows how the MglA data are linked to the SUPERFAMILY database in the RDF graph (see also Figure 9). PSN identifiers used in the MglA data are connected to FTN identifiers. Superfamily annotations are linked via PID identifiers which are connected to the FTN identifiers.

### Querying combined experiments

Here we give example queries for the proteomics and the transcriptomics data sets. Since these data were already published, we chose to use the integrated data to perform some verification of the experimental results published in [5] and [50]. We took the 10 up-regulated genes according to [6] and compared the transcriptomic results with the proteomics data, and in the final example, we took two genes from the proteomics data and gathered the transcriptomic data.

#### Proteomics data query: mutant Vs wildtype

The query in Table 7 exemplifies a typical query (from the experimental design) that would be performed over the proteomics data. This query returns a list of Fn locus tags observed to be highly abundant in the mutant and not present (abundance of 0) in the wildtype. This query displays the internal integration of this data set. These data were originally in three separate spreadsheets but were brought together into RDF and can now be queried as a unit.

**Table 7:** Proteomics Data Combination Query. Proteomics data query: mutant Vs wildtype The query in Table 7 exemplifies a typical query (from the experimental design) that would be performed over the proteomics data. This query returns a list of Fn locus tags observed to be highly abundant (z = 100,000) in the mutant and not present (z = 0) in the wildtype. This query displays the internal integration of this data set. These data were original in three separate spreadsheets but were brought together into RDF and can now be queried as a a unit. Query 1. SeRQL select query identifies FTN identifiers for proteins that were highly abundant in the mutant and with no presence in the wildtype.

SELECT distinct ftn FROM
**{ftt} ncbi:blastn {ftn},**
**{psn} rdfs:seeAlso {ftn**}**,**
**{analysis} mgla:poson {psn},**
**{analysisID} rdf:subject {analysis},**
**{analysisID} rdf:object {exp},**
**{analysisID} mgla:PeptideAbundance {z}**
WHERE xsd:integer(z) > 100000
AND exp LIKE "*mutant*"
INTERSECT
SELECT ftn FROM
**{ftt} ncbi:blastn {ftn},**
**{psn} rdfs:seeAlso {ftn},**
**{analysis} mgla:poson {psn},**
**{analysisID} rdf:subject {analysis},**
**{analysisID} rdf:object {exp},**
**{analysisID} mgla:PeptideAbundance {z}**
WHERE xsd:integer(z) = 0
AND exp LIKE "*wildtype*"
USING namespace
mgla = <http://www.francisella.org/novicida/schema/fnu112/experiments/mgla/>,
soft = <http://www.ncbi.nlm.nih.gov/projects/geo/info/soft2.html#>,
nwrce = <https://tools.nwrce.org/geo/schema/>,
ncbi = <http://ncbi.nlm.nih.gov/>.

#### Transcriptomic data query

The query given in Table 8 returns, side by side, the transcript abundance values for genes in the wildtype and in the mutant across all samples. Part of the results of this query are shown in Table 9 from which we can see the difference between wildtype abundance and mutant abundance for the locus tag FTT_0551.

**Table 8:** Transcriptomic Experiment Data Combination Query. Query 2. SeRQL comparison of wildtype samples and mutant samples for the gene with locus tag

FTT 0551.
SELECT ftt, Source2, phase, x, Source1, phase2, v FROM
**{ftt} ncbi:blastn {ftn},**
**{ftn} nwrce:OligoID {oligo},**
**{platform} soft:array_oligo_id {oligo},**
**{sample} mgla:SpotID {platform},**
**{sample} soft:sample_value {v},**
**{sampleID} soft:Sample_data_table_id {sample},**
**{sampleID} mgla:SampleSource {Source1},**
**{sampleID} mgla:SampleTimepoint_source {phase},**
**{sample2} mgla:SpotID {platform},**
**{sample2} soft:sample_value {x},**
**{sampleID2} soft:Sample_data_table_id {sample2},**
**{sampleID2} mgla:SampleSource {Source2},**
**{sampleID2} mgla:SampleTimepoint_source {phase2}**
WHERE ftt = <http://www.genome.jp/dbget-bin/www_bget?ftu+FTT0551>
AND Source1 != Source2
AND Source1 = Mutant
AND phase = phase2
AND phase2 = 1st growth curve
USING namespace
mgla = <http://www.francisella.org/novicida/schema/fnu112/experiments/mgla/>,
soft = <http://www.ncbi.nlm.nih.gov/projects/geo/info/soft2.html#>,
nwrce = <https://tools.nwrce.org/geo/schema/<,
ncbi = <http://ncbi.nlm.nih.gov/>.

**Table 9:** Transcriptomic Experiment Data Combination Results. Partial results from Query 2 (Table 8) showing that the locus tag FTT_0551 is upregulated in the mutant.

locus tag	set 1		Abundance value	set 2		Abundance Value
FTT0551	Wild Type	1st growth curve	**.292**	Mutant	1st growth curve	**-.855**
FTT0551	Wild Type	1st growth curve	**-.381**	Mutant	1st growth curve	**-.855**
FTT0551	Wild Type	1st growth curve	**.292**	Mutant	1st growth curve	**1.691**
FTT0551	Wild Type	1st growth curve	**-.381**	Mutant	1st growth curve	**1.691**
FTT0551	Wild Type	1st growth curve	**.796**	Mutant	1st growth curve	**-1.079**
FTT0551	Wild Type	1st growth curve	**.541**	Mutant	1st growth curve	**-1.079**
FTT0551	Wild Type	1st growth curve	**.796**	Mutant	1st growth curve	**1.481**
FTT0551	Wild Type	1st growth curve	**.541**	Mutant	1st growth curve	**1.481**

#### Verification queries

Once data are integrated and queryable together more information is available through comparison of the data. For example, we are able to use these integrated data sets to verify that the genes that were found to be up regulated in the transcriptomics experiment were also up-regulated in the proteomics experiment. An example query for the locus tag FTT_0552 is shown in Table 10. The 10 up-regulated genes in the MglA mutant are given in Table 1 of [6], the verification results are shown in Table 11 below. From this table we see that proteomics abundance differences between wildtype and mutant were only available for 5 out of the 10 genes. The locus tags FTT0747, FTT1288, FTT0844, FTT1529 and FTT1532 gave abundance values of 0 across the wildtype and mutant samples. Although no statistical analyses are performed here, we do see that upregulation at the peptide level was only seen in 3 of 5 of the genes. Through the very simple analysis performed here, it would be impossible to make any conclusive statements regarding the correlation between peptide abundance and transcript abundance, however, the availability of the data integrated in this form should enable a more sophisticated analysis of these two experiments.

**Table 10:** Verification Query comparing experimental results for the top 10 upregulated genes. Query 3. SeRQL query selects wildtype peptide abundance for locus_tag FTT_0552, upregulated in the transcriptomic experiment.

SELECT ftt, ftn, exp, x FROM
**{ftt} ncbi:blastn {ftn},**
**{psn} rdfs:seeAlso {ftn},**
**{analysis} mgla:poson {psn},**
**{analysisID} rdf:subject {analysis},**
**{analysisID} rdf:object {exp},**
**{analysisID} mgla:PeptideAbundance {x}**
WHERE ftt = <http://www.genome.jp/dbget-bin/www_bget?ftu+FTT0552>
AND xsd:integer(x) > 0
AND exp LIKE "*wildtype*"
USING namespace mgla = <http://www.francisella.org/novicida/schema/fnu112/experiments/mgla>,
soft = <http://www.ncbi.nlm.nih.gov/projects/geo/info/soft2.html#>,
nwrce = <https://tools.nwrce.org/geo/schema/>,
ncbi = <http://ncbi.nlm.nih.gov/>

**Table 11:** Verification Query Results for the top 10 upregulated genes. This table gives the 10 upregulated genes in the transcriptomics experiment with the mean peptide abundance data for the wildtype and mutant samples from the proteomics experiment.

locus tag	fold change	Wt abundance (mean)	Mt abundance (mean)	difference
FTT0551	2.11	0	5057	+5057
FTT0552	2.01	10463	18428	+7965
FTT0553	1.97	28075	38517	+10442
FTT0747	1.70	0	0	0
FTT0844	1.59	0	0	0
FTT1195	2.64	26067	21361	-4706
FTT1288	1.41	0	0	0
FTT1529	1.20	0	0	0
FTT1532	0.98	0	0	0
FTT1741	1.67	61070	15574	-45495

## Discussion

### Unique Identifiers

URIs, Uniform Resource Identifiers, are the base concept on which the semantic web technologies were developed. All things on the semantic web are resources, and all resources may be identified by URIs. The use of globally unique identification (GUID) can greatly facilitate data integration [51]. For example, when two data nodes are the same in two resources, those data can be reconciled very easily if the nodes use GUIDs. In bioinformatics, the databases Genbank and EMBL share a unique identifier called an Accession number. A user can use this identifier to retrieve the same sequence in either database. This also means that this unique identifier can be used to reconcile these sequences if two separate resources make reference to the same sequence. If the unique identifier is used, we know that both resources are referring to the same sequence. Where individual data sources use their own forms of unique identification, a URI can make those identifiers unique, for example, http://www.protein.org/seq#123456 and http://www.gene.org/seq#123456. The use of URIs for unique identification can resolve the issue of the same identifier used in different databases to refer to different things.

Lack of persistent unique identification in various Fn data sets meant that the proteomics experiment and the transcriptomic experiment could not be combined. However, by combining data in RDF, the different identifiers used in the Fn data have been reconciled and the RDF graph can be used as a source for cross references from the experimental data and the annotation in public domain data sources. However, in the long term, for semantic web approaches to be successful in biology, *data producers and users need supported tools that can produce and resolve persistent unique identifiers*.

### XML data exchange formats

The bioinformatics community have invested heavily in data exchange formats in XML. There are numerous examples. MIAME [52] is a standard format for microarray experiments. The Proteomics Standards Initiative http://www.psidev.info/ have developed MIAPE for proteomics mass spectrometry data and other standard exchange formats for chromatography and gel electrophoresis. Data interchanged in standard formats like these can be readily transformed into RDF. These formats can also be used as the predicate vocabulary. Wherever possible, it was our aim to use a standard term, when a suitable one existed. Currently, standard, easily accessible vocabularies are lacking. This has a lot to do with the fact that the omics XML standards were built as data exchange formats and *using them as vocabularies is out of scope*. However, our experience highlights that further work is required in this area and some further coordination and extension of vocabularies and checklists is required.

We created simple XSLT scripts to convert data from these standard formats into RDF. Conversion scripts from common data formats such as FASTA and GenBank were created using Perl. These scripts are far easier to develop and more readily reusable than the traditional data warehouse ETL processes, and this mechanism of data interchange is more accessible to biologists.

Standard vocabulary terms can also facilitate data integration. For example, just as two nodes that share the same URI are resolved, nodes in different graphs may be linked together by shared predicates. We required and ultimately created a vocabulary in RDF-S that described the experimental design of the MglA mutant experiment in order to easily integrate the peptide abundance data with the standard protein identification data that was in the ProtXML format. Although this paper focuses on integration at the level of resource identifiers, *further integration can be achieved via combing MglA data and the protein identifications at the level of properties used in both RDF graphs*.

### Annotation of data analysis results

We found that experimental procedures and raw data are easily accessible in standard representations, however, analysed data, such as those found in secondary databases and published in papers are generally only available in ad hoc formats and on journal web pages. While progress has been made in standardisation of experimental data, *the analysis process and the analysed data still require an exchange standard*. This task might be handled partly by work flow descriptions and by standard vocabularies.

## Conclusion

This paper demonstrates the progress made while testing semantic web technologies for data integration and highlights gaps and further requirements in data integration support. We demonstrated that data integration using RDF is easy to carry out and that simple integration at the level of resource identifiers can be achieved cheaply and efficiently. The combined data in the RDF graph provides a resource for database cross references for Fn data which enabled the integration of two experimental data sets. This resource increases the depth of annotation available to biologists and this form of integration reduces the manual effort that would normally be required to gain this depth of annotation.

The integration of experimental data offers the opportunity to compare and verify the results from different omics technologies. Through this prototype we were able to perform queries across the data to compare and contrast the experimental observations. The advantages that integration of experimental data offers are shown above in the queries that were made possible over the integrated data. Although it would be impossible to make any conclusive statements from the simple analysis performed here, with respect to correlation between peptide abundance and transcript abundance, the availability of the data in this integrated form should enable sophisticated analysis of these two experiments such as performed by [53], and indeed this would be the next step in the analysis of these data. Also, the integration performed with these experiment data and the public domain annotations has enabled us to query both experiments with respect to specific KEGG pathways and GO categories which proved to be particularly helpful. We were able, for example, to extract genes that could not be related to any data source and that displayed particular transcription patterns in the mutant at both the transcriptomic and proteomic level, which prompted us to look for similar transcriptional patterns in known biological pathways. These genes may form novel pathways within this organism that can now be further investigated in the laboratory.

We found that using RDF and RDF-S to combine data is a more timely and responsive solution for this kind of data integration, as compared to traditional integration methods. Building boutique integration systems with warehouse or federated approaches is not cost effective for one off comparisons of experimental data. We demonstrated that the development of this boutique system using semantic web technologies has provided data for further investigation at a considerably lower cost and more timely than a traditional approach would have afforded. Development effort was placed in parsing data into RDF and querying the data. Since data of this nature are predominantly stored in spreadsheets, from which RDF triples are easily created, there was little 'data modelling' performed. Using semantic web technologies, there is no 'global schema' design phase, or extraction, transformation, and finally no data are loaded into a 'global schema', therefore avoiding the time consuming data mapping process. The RDF-S was built incrementally as further data sources were added. However, the complexity of the combined data is reflected in the graph and the queries over the graph. The data were combined based on shared identifiers in the different data sets and RDF-S was used to combine the data from the experimental fractions of the proteomics data. The development of the RDF-S file was the most time consuming portion of this project. In conclusion, since there was no requirement to remodel or restructure each data source into the same 'global structure', the prototype contained queryable data within only a few days of effort. This effort was spent on the building the data transformation scripts which were mostly Perl and XSLT. Generally, the data were transformed into RDF using their native data models, so no traditional 'modelling' time was required (for example, the GEO data are modelled into Profile, Series and Samples and we used exactly this model for the RDF). Additionally, the use of RDF and RDF-S for data combination offered considerable flexibility. For example, the addition of data was a simple process of transforming the data into RDF and loading it into the repository. In a typical warehouse solution, an additional data source often requires changes to the 'global schema'. We were able to add and remove data with ease and with no impact to queries over the rest of the data. Also, the approach used is faster to develop and re-usable. The RDF file for each data set are available for download and a full repository dump is also provided. These files will enable other users to combine these data with their own RDF data. Many projects now provide data in RDF and it is very easy with these technologies to re-use these data in other integration mashups. We found the technologies had some limitations though. For example, the SeRQL query language lacks aggregate functions (MIN, MAX, MEAN etc.) and useful features such as 'order by' and 'group by' which are available in SQL. However, we feel that these limitations did not effect the data integration requirements of this prototype and since these can be overcome with ease in other applications we were not greatly affected. We anticipate that once these technologies are used more widely, these features will be available in future releases.

We conclude that these technologies offer a cost effective and viable solution for "one off" integration requirements in biology as exemplified here for two experimental data sets. We hope that in the future datasets will be published in XML or RDF formats to support more flexible data integration.

## Availability and requirements

A repository dump in Ntriple format can be downloaded from http://spira.bio.gla.ac.uk/SWAT4LSBMC/

## Electronic supplementary material

Additional file 1: **MglA Parse Script**. Perl Script used to parse the MglA wholecell data. (PDF 47 KB)

Additional file 2: **GEO Parse Script**. Perl Script used to parse the Geo data. (PDF 54 KB)

## List of abbreviations

Ft: *Francisella tularensis*
Fn: *Francisella tularensis novicida*
FPI: Francisella Pathogenecity Island
MglA: Macrophage growth locus A
RDF: Resource Description Framework
RDF-S: Resource Description Framework (RDF) Schema
OWL: Web Ontology Language
XSLT: Extensible Stylesheet Language Transformation
SeRQL: Sesame Rdf Query Language
SQL: Structured Query Language.
