# Supplementary material for: Francisella tularensis novicida proteomic and transcriptomic data integration and annotation based on semantic web technologies
Source: BMC Bioinformatics. 2009 Oct 1;10(Suppl 10):S3. doi: 10.1186/1471-2105-10-S10-S3 (PMC2755824; doi:10.1186/1471-2105-10-S10-S3)
Supplement: Supplementary file 1 — Additional file 1: MglA Parse Script. Perl Script used to parse the MglA wholecell data. (PDF 47 KB) [file 12859_2009_3371_MOESM1_ESM.pdf]

```
#!/usr/bin/perl

# Nadia Anwar

# Seattle January 2008


# Input file MGLA - csv


# Output file RDF/XML

# Open file Handles

open (CSV, "WholeCell_Lvl7_02_protein_function_tg.txt"|| die);

open (RDF, "+>WholeCellReified_3.rdf");

# RDF XML Header

print RDF "<?xml version=\"1.0\" encoding=\"UTF-8\"?>\n";

print RDF "<rdf:RDF";

print RDF "
xml:base=\"http://www.francisella.org/novicida/schema/fnu112/experiments/mgla/\"\n";

print RDF " xmlns:rdf=\"http://www.w3.org/1999/02/22-rdf-syntax-ns#\"\n";

print RDF "\t xmlns:rdfs=\"http://www.w3.org/2000/01/rdf-schema#\"\n";

print RDF "\t xmlns:owl=\"http://www.w3.org/2002/07/owl#\" \n";

print RDF "\t
xmlns:mgla=\"http://www.francisella.org/novicida/schema/fnu112/experiments/mgla/\"\n";

print RDF "\t xmlns:protXML=\"http://regis-web.systemsbiology.net/protXML/\"\n";

print RDF "\t
xmlns:fnu112=\"http://www.francisella.org/novicida/schema/fnu112/experiments/mgla#\">\n";


# slurp data from file handle

@data=<CSV>;

%posons="";

$line_number=0;

foreach $line (@data){

$line_number++;

$line =~ s/"//;

# build array to shift

@data_elements =split("\t", $line);

$peptide = shift(@data_elements);

$abundance01_wc_01 = shift(@data_elements);
```

```
$abundance01_wc_02 = shift(@data_elements);

$abundance01_wc_03 = shift(@data_elements);

$abundance01_wc_04 = shift(@data_elements);

$abundance11_wc_01 = shift(@data_elements);

$abundance11_wc_02 = shift(@data_elements);

$abundance11_wc_03 = shift(@data_elements);

$abundance11_wc_04 = shift(@data_elements);

$pval=shift(@data_elements);

$pval1=shift(@data_elements);

$psn=shift(@data_elements);

$description=shift(@data_elements);

$cog=shift(@data_elements);

$go=shift(@data_elements);

$go2=shift(@data_elements);

$location=shift(@data_elements);

chomp($location);


$psnannotation = $description."\t".$cog."\t".$go."\t".$go2."\t".$location;

unless ( grep {$_ eq $psn} (keys %posons) ) {

$posons{$psn} = $psnannotation;

}


# defined namesspaces

$dataset="WholeCell_Lvl7_02";

$mgla="http://www.francisella.org/novicida/schema/fnu112/experiments/mgla";

$fnu112_schema="http://www.francisella.org/novicida/schema/fnu112/experiments/mgla#";



# Peptide triples starts here

print RDF "<mgla:IdentifiedPeptide rdf:about=\"$mgla/$dataset.$line_number\">\n";

print RDF "\t <mgla:pval>$pval</mgla:pval>\n";

print RDF "\t <mgla:pval1>$pval1</mgla:pval1>\n";

print RDF "\t <mgla:PeptideSequence>$peptide</mgla:PeptideSequence>\n";
```

```
# Reified triple

print RDF "\t<mgla:InExperimentReplicate rdf:resource=\"$mgla/wildtype/01_wc_01\"
rdf:ID=\"$dataset.$line_number".($line_number+1). "\" />\n";

# Reified triple

print RDF "\t<mgla:InExperimentReplicate rdf:resource=\"$mgla/wildtype/01_wc_02\"
rdf:ID=\"$dataset.$line_number".($line_number+2). "\" />\n";

# Reified triple

print RDF "\t<mgla:InExperimentReplicate rdf:resource=\"$mgla/wildtype/01_wc_03\"
rdf:ID=\"$dataset.$line_number".($line_number+3). "\" />\n";

# Reified triple

print RDF "\t<mgla:InExperimentReplicate rdf:resource=\"$mgla/wildtype/01_wc_04\"
rdf:ID=\"$dataset.$line_number".($line_number+4). "\" />\n";

# Reified triple

print RDF "\t<mgla:InExperimentReplicate rdf:resource=\"$mgla/mutant/11_wc_01\"
rdf:ID=\"$dataset.$line_number".($line_number+13). "\" />\n";

# Reified triple

print RDF "\t<mgla:InExperimentReplicate rdf:resource=\"$mgla/mutant/11_wc_02\"
rdf:ID=\"$dataset.$line_number".($line_number+14). "\" />\n";

# Reified triple

print RDF "\t<mgla:InExperimentReplicate rdf:resource=\"$mgla/mutant/11_wc_03\"
rdf:ID=\"$dataset.$line_number".($line_number+15). "\" />\n";

# Reified triple

print RDF "\t<mgla:InExperimentReplicate rdf:resource=\"$mgla/mutant/11_wc_04\"
rdf:ID=\"$dataset.$line_number".($line_number+16). "\" />\n";

if ($psn ne ""){

print RDF "\t<mgla:poson
rdf:resource=\"https://tools.nwrce.org/cgi-bin/fnu112/poson.cgi?poson=$psn\"/>\n";

}

print RDF "</mgla:IdentifiedPeptide>\n";

# Reified abundance triples start here

print RDF "\t<rdf:Description
rdf:about=\"#$dataset.$line_number".($line_number+1)."\"\>\n";

print RDF "\t\t <mgla:PeptideAbundance>$abundance01_wc_01</mgla:PeptideAbundance>\n";

print RDF "\t</rdf:Description>\n";



print RDF "\t<rdf:Description
rdf:about=\"#$dataset.$line_number".($line_number+2)."\"\>\n";

print RDF "\t\t <mgla:PeptideAbundance>$abundance01_wc_02</mgla:PeptideAbundance>\n";

print RDF "\t</rdf:Description>\n";
```

```
print RDF "\t<rdf:Description
rdf:about=\"#$dataset.$line_number".($line_number+3)."\"\>\n";

print RDF "\t\t <mgla:PeptideAbundance>$abundance01_wc_03</mgla:PeptideAbundance>\n";

print RDF "\t</rdf:Description>\n";



print RDF "\t<rdf:Description
rdf:about=\"#$dataset.$line_number".($line_number+4)."\"\>\n";

print RDF "\t\t <mgla:PeptideAbundance>$abundance01_wc_04</mgla:PeptideAbundance>\n";

print RDF "\t</rdf:Description>\n";



print RDF "\t<rdf:Description
rdf:about=\"#$dataset.$line_number".($line_number+13)."\"\>\n";

print RDF "\t\t <mgla:PeptideAbundance>$abundance11_wc_01</mgla:PeptideAbundance>\n";

print RDF "\t</rdf:Description>\n";



print RDF "\t<rdf:Description
rdf:about=\"#$dataset.$line_number".($line_number+14)."\"\>\n";

print RDF "\t\t <mgla:PeptideAbundance>$abundance11_wc_02</mgla:PeptideAbundance>\n";

print RDF "\t</rdf:Description>\n";



print RDF "\t<rdf:Description
rdf:about=\"#$dataset.$line_number".($line_number+15)."\"\>\n";

print RDF "\t\t <mgla:PeptideAbundance>$abundance11_wc_03</mgla:PeptideAbundance>\n";

print RDF "\t</rdf:Description>\n";



print RDF "\t<rdf:Description
rdf:about=\"#$dataset.$line_number".($line_number+16)."\"\>\n";

print RDF "\t\t <mgla:PeptideAbundance>$abundance11_wc_04</mgla:PeptideAbundance>\n";

print RDF "\t</rdf:Description>\n";

}#foreach

# Annotation triples

foreach $poson (keys %posons){

if ($poson ne ""){

print RDF "\t <rdf:Description
rdf:about=\"https://tools.nwrce.org/cgi-bin/fnu112/poson.cgi?poson=$poson\">\n";

$annotationvalue=$posons{$poson};
```

```
@annotations = split("\t", $annotationvalue);

$description=shift(@annotations);

$cog=shift(@annotations);

$go=shift(@annotations);

$go2=shift(@annotations);

$location=shift(@annotations);



print RDF "\t <mgla:annotaton>$description</mgla:annotaton>\n";

print RDF "\t <mgla:localization>$localization</mgla:localization>\n";



if ($cog ne ""){

$cog =~ s/"//g;

@cog_data = split(", ", $cog);

$cog_number=shift(@cog_data);

$cog_number=~s/"//g;

print RDF "\t <mgla:cogNumber ";

print RDF
"rdf:resource=\"http://www.ncbi.nlm.nih.gov/sites/entrez?db=cdd&amp;cmd=search&amp;term=$
cog_number\"/>\n";

$cog_domain_name=shift(@cog_data);

print RDF "\t <mgla:cogDomain>";

print RDF "$cog_domain_name";

print RDF "</mgla:cogDomain>\n";

$cog_description=shift(@cog_data);

print RDF "\t <mgla:cogDescription>";

print RDF "$cog_description";

print RDF "</mgla:cogDescription>\n";

$cog_category=shift(@cog_data);

print RDF "\t <mgla:cogCategory>";

print RDF "$cog_category";

print RDF "</mgla:cogCategory>\n";

#print RDF "\t </mgla:cogNumber>\n";

}
```

```
if ($go ne ""){

$go =~ s/"//g;

@godata = split("-", $go);

$go_ID = shift(@godata);

$go_DES = shift(@godata);

print RDF "\t <mgla:goAnnotation ";

print RDF
"rdf:resource=\"http://amigo.geneontology.org/cgi-bin/amigo/term-details.cgi?term=$go_ID\
"/>\n";

print RDF "\t <mgla:goDescription>$go_DES</mgla:goDescription>\n";

#print RDF "\t </mgla:goAnnotation>\n";

}

if ($go2 ne ""){

$go2 =~ s/"//g;

@go2data = split("-", $go2);

$go2_ID = shift(@go2data);

$go2_DES = shift(@go2data);

print RDF "\t <mgla:goAnnotation ";

print RDF
"rdf:resource=\"http://amigo.geneontology.org/cgi-bin/amigo/term-details.cgi?term=$go2_ID
\"/>\n";

print RDF "\t <mgla:goDescription>$go2_DES</mgla:goDescription>\n";

#print RDF "\t </mgla:goAnnotation>\n";

}



print RDF "\t</rdf:Description>\n";

}

}



print RDF "</rdf:RDF>\n";
```
