# Supplementary material for: Francisella tularensis novicida proteomic and transcriptomic data integration and annotation based on semantic web technologies
Source: BMC Bioinformatics. 2009 Oct 1;10(Suppl 10):S3. doi: 10.1186/1471-2105-10-S10-S3 (PMC2755824; doi:10.1186/1471-2105-10-S10-S3)
Supplement: Supplementary file 2 — Additional file 2: GEO Parse Script. Perl Script used to parse the Geo data. (PDF 54 KB) [file 12859_2009_3371_MOESM2_ESM.pdf]

```
#!/usr/bin/perl

# GEO_family_soft_parse.pl

# Nadia Anwar

# Seattle August 2008


# Input file GEO - soft

# ftp.ncbi.nih.gov/pub/geo/DATA/SOFT/by_series/GSE5468/


# Output file RDF/XML


$file2parse = $ARGV[0];

if (!$file2parse){

 die "usage: ./GEO_family_soft_parse.pl <filename>\n";

}


open (TXT, "$file2parse") || die "\t can't open file: $file2parse\n";

open (RDF, "+>$file2parse.rdf");

# RDF XML Header

print RDF "<?xml version=\"1.0\" encoding=\"UTF-8\"?>\n";

print RDF "<rdf:RDF";

print RDF " xml:base=\"http://www.ncbi.nlm.nih.gov/projects/geo/\"\n";

print RDF " xmlns:rdf=\"http://www.w3.org/1999/02/22-rdf-syntax-ns#\"\n";

print RDF "\t xmlns:rdfs=\"http://www.w3.org/2000/01/rdf-schema#\"\n";

print RDF "\t xmlns:owl=\"http://www.w3.org/2002/07/owl#\" \n";

print RDF "\t
xmlns:soft=\"http://www.ncbi.nlm.nih.gov/projects/geo/info/soft2.html#\">\n";

$base="http://www.ncbi.nlm.nih.gov/projects/geo";

@data=<TXT>;

get_series(); # Run routine to get SERIES data

# get file location for Platform table data and Sample table data

foreach $record (@data){

$linecount++;

if ($record =~ m/!platform_table_begin/){

$tableBegin=$linecount;
```

```
#print "Platfrom table begins on line number: $tableBegin\n";

}


if ($record =~ m/!platform_table_end/){

$tableEnd=$linecount;

$tableLength = $tableEnd-$tableBegin;

#print "Platform table ends on line number: $tableEnd\n table length = $tableLength\n";

}


if ($record =~ m/\^SAMPLE = (\S+)/){

$sampleDataID = $1;

$SampleRecordBegin = $linecount;

push @sampleRecords, $SampleRecordBegin;

}


if ($record =~ m/!sample_table_end/){

$SampletableEnd=$linecount;

$Sampletablelength = $SampletableEnd-$SampleRecordBegin;

#print "Sample table ends on line number: $SampletableEnd\n table length = $Sampletable-
length\n";

push @sampleRecordLengths, $Sampletablelength;

}

@samples{ @sampleRecords } = @sampleRecordLengths;

}

@platformtable=@data;

get_platform(); # Run routine to get PLATFORM data

@sampletable=@data;

get_sample(); # Run routine to get SAMPLE data

print RDF "</rdf:RDF>\n";


# Subroutines definitions start here

sub get_series {

foreach $record (@data){

chomp($record);
```

```
if ($record =~ m/\^SERIES = (\S+)/){

$seriesID = $1;

print RDF "<soft:Series
rdf:about=\"http://www.ncbi.nlm.nih.gov/geo/query/acc.cgi?acc=$seriesID\">\n";

}



if ($record =~ m/Series_platform_id = (.*)/){

print RDF "<soft:Series_platform_id
rdf:resource=\"http://www.ncbi.nlm.nih.gov/geo/query/acc.cgi?acc=$1\"/>\n";

}



if ($record =~ m/!Series_sample_id = .*/){

#print $record;

$sampleid = $record;

$sampleid =~ m/!Series_sample_id = (.*)/;

$sampleid = $1;

print RDF "<soft:Series_sample_id
rdf:resource=\"http://www.ncbi.nlm.nih.gov/geo/query/acc.cgi?acc=$sampleid\"/>\n";

}



if ($record =~ m/\!Series_title = (.*)$/){

$seriesTitle=$1;

print RDF "<soft:Series_title>$seriesTitle</soft:Series_title>\n";

}



if ($record =~ m/\!Series_status = (.*)$/){

$seriesStatus = $1;

print RDF "<soft:Series_status>$seriesStatus</soft:Series_status>\n";

}



if ($record =~ m/!Series_submission_date = (.*)/){

$seriesSubDate =$1;

print RDF "<soft:Series_submission_date>$seriesSubDate</soft:Series_submission_date>\n";

}
```

```
if ($record =~ m/!Series_last_update_date = (.*)/){

$seriesUpdate = $1;

print RDF "<soft:Series_last_update_date>$seriesUpdate</soft:Series_last_update_date>\n";

}


if ($record =~ m/!Series_pubmed_id = (.*)/){

$seriesPubmed = $1;

print RDF "<soft:Series_pubmed_id>$seriesPubmed</soft:Series_pubmed_id>\n";

}


if ($record =~ m/!Series_summary = (.*)/){

$seriesSummary = $1;

print RDF "<soft:Series_summary>$seriesSummary</soft:Series_summary>\n";

}


if ($record =~ m/!Series_summary = Keyword: (.*)/){

$seriesSummary = $1;

print RDF "<soft:Series_summary_keyword>$seriesSummary</soft:Series_summary_keyword>\n";

}


if ($record =~ m/!Series_overall_design =(.*)/){

$seriesDesign = $1;

print RDF "<soft:Series_overall_design>$seriesDesign</soft:Series_overall_design>\n";

}


if ($record =~ m/!Series_contributor = (.*)/){

$seriesCon = $1;

print RDF "<soft:Series_contributor>$seriesCon</soft:Series_contributor>\n";

}

}

print RDF "</soft:Series>\n";

# end of series resource
```

```
}


sub get_platform {

$linecount=0;

foreach $record (@data){

$linecount++;

chomp($record);


if ($record =~ m/\^PLATFORM = (\S+)/){

$platformID = $1;

#print $record;

print RDF "<soft:Platform
rdf:about=\"http://www.ncbi.nlm.nih.gov/geo/query/acc.cgi?acc=$platformID\">\n";

}


if ($record =~ m/!Platform_title = (.*)?/){

print RDF "<soft:Platform_title>$1</soft:Platform_title>\n";

}


if ($record =~ m/!Platform_status = (.*)/){

print RDF "<soft:Platform_status>$1</soft:Platform_status>\n";

}


if ($record =~ m/!Platform_submission_date = (.*)/){

print RDF "<soft:Platform_submission_date>$1</soft:Platform_submission_date>\n";

}


if ($record =~ m/!Platform_last_update_date = (.*)/){

print RDF "<soft:Platform_last_update_date>$1</soft:Platform_last_update_date>\n";

}


if ($record =~ m/!Platform_technology = (.*)/){

print RDF "<soft:Platform_technology>$1</soft:Platform_technology>\n";

}
```

```
if ($record =~ m/!Platform_distribution = (.*)/){

print RDF "<soft:Platform_distribution>$1</soft:Platform_distribution>\n";

}


if ($record =~ m/!Platform_organism = (.*)/){

print RDF "<soft:Platform_organism>$1</soft:Platform_organism>\n";

}


if ($record =~ m/!Platform_manufacturer = (.*)/){

print RDF "<soft:Platform_manufacturer>$1</soft:Platform_manufacturer>\n";

}


if ($record =~ m/!Platform_manufacture_protocol = (.*)/){

print RDF
"<soft:Platform_manufacturer_protocol>$1</soft:Platform_manufacturer_protocol>\n";

}


if ($record =~ m/!Platform_support = (.*)/){

print RDF "<soft:Platform_support>$1</soft:Platform_support>\n";

}


if ($record =~ m/!Platform_coating = (.*)/){

print RDF "<soft:Platform_coating>$1</soft:Platform_coating>\n";

}


if ($record =~ m/!Platform_contact_name = (.*)/){

print RDF "<soft:Platform_contact_name>$1</soft:Platform_contact_name>\n";

}


if ($record =~ m/!Platform_contact_email = (.*)/){

print RDF "<soft:Platform_contact_email>$1</soft:Platform_contact_email>\n";

}
```

```
if ($record =~ m/!Platform_data_row_count = (.*)/){
print RDF "<soft:Platform_data_row_count>$1</soft:Platform_data_row_count>\n";
}
}

# Parse Platform Table
@table = splice(@platformtable, $tableBegin, $tableLength);
shift(@table)."\n";
pop(@table)."\n";
foreach $tablerow (@table){
#print "**\t$tablerow\n";
@tablerowdata = split("\t", $tablerow);
$T_ID = $tablerowdata[0];
print RDF "<soft:Platform_data_table_id rdf:resource=\"$base/$platformID.$T_ID\"/>\n";
}

print RDF "</soft:Platform>\n";

foreach $tablerow (@table){
@rowdata = split("\t", $tablerow);
$T_ID = $rowdata[0];
$T_MC = $rowdata[1];
$T_MR = $rowdata[2];
$T_COLUMN = $rowdata[3];
$T_ROW = $rowdata[4];
$T_ORF = $rowdata[5];
$T_OLIGOID = $rowdata[6];
$T_CONTROL =$rowdata[7];
$T_POLYMER = $rowdata[8];
$T_TYPE = $rowdata[9];
$T_SPOTID = $rowdata[10];

print RDF "<soft:Platform_data_table rdf:about=\"$base/$platformID.$T_ID\"\>\n";
print RDF "\t <soft:array_column>$T_COLUMN</soft:array_column>\n";
```

```
print RDF "\t <soft:array_row>$T_ROW</soft:array_row>\n";

print RDF "\t <soft:array_orf>$T_ORF</soft:array_orf>\n";

print RDF "\t <soft:array_oligo_id>$T_OLIGOID</soft:array_oligo_id>\n";

print RDF "\t <soft:array_control>$T_CONTROL</soft:array_control>\n";

print RDF "\t <soft:array_polymer>$T_POLYMER</soft:array_polymer>\n";

print RDF "\t <soft:array_type>$T_TYPE</soft:array_type>\n";

print RDF "\t <soft:array_spot_id>$T_SPOTID</soft:array_spot_id>\n";

print RDF "</soft:Platform_data_table>\n";

}


}# sub get_platform


sub get_sample {

#Parse Sample Data

while ( my ($key, $value) = each(%samples) ) {

$cnt=0;

#print "$key => $value\n";

@samplesplice = @data;

@SampleRecord=splice(@samplesplice, $key-1,$value);

@Sampletablesplice=@SampleRecord;

foreach $SampleRecord (@SampleRecord){

$cnt++;

#print "$SampleRecord***\n";

if ($SampleRecord =~ m/\^SAMPLE = (\S+)/){

$sampleResource = $1;

print RDF "<soft:Sample
rdf:about=\"http://www.ncbi.nlm.nih.gov/geo/query/acc.cgi?acc=$sampleResource\"/>\n";

}


if ($SampleRecord =~ m/!Sample_title = (.*)/){

print RDF "<soft:Sample_title>$1</soft:Sample_title>\n";

}


if ($SampleRecord =~ m/!Sample_geo_accession = (.*)/){
```

```
print RDF "<soft:Sample_geo_accession
rdf:resource=\"http://www.ncbi.nlm.nih.gov/geo/query/acc.cgi?acc=$1\"/>\n";

}



if ($SampleRecord =~ m/!Sample_status = (.*)/){

print RDF "<soft:Sample_status>$1</soft:Sample_status>\n";

}



if ($SampleRecord =~ m/!Sample_submission_date = (.*)/){

print RDF "<soft:Sample_submission_date>$1</soft:Sample_submission_date>\n";

}



if ($SampleRecord =~ m/!Sample_last_update_date = (.*)/){

print RDF "<soft:Sample_last_update_date>$1</soft:Sample_last_update_date>\n";

}



if ($SampleRecord =~ m/!Sample_type = (.*)/){

print RDF "<soft:Sample_type>$1</soft:Sample_type>\n";

}



if ($SampleRecord =~ m/!Sample_channel_count = (.*)/){

print RDF "<soft:Sample_channel_count>$1</soft:Sample_channel_count>\n";

}



if ($SampleRecord =~ m/!Sample_source_name_ch1 = (.*)/){

print RDF "<soft:Sample_source_name_ch1>$1</soft:Sample_source_name_ch1>\n";

}



if ($SampleRecord =~ m/!Sample_organism_ch1 = (.*)/){

print RDF "<soft:Sample_organism_ch1>$1</soft:Sample_organism_ch1>\n";

}



if ($SampleRecord =~ m/!Sample_characteristics_ch1 = (.*)/){

print RDF "<soft:Sample_characteristics_ch1>$1</soft:Sample_characteristics_ch1>\n";
```

```
}

if ($SampleRecord =~ m/!Sample_molecule_ch1 = (.*)/){
print RDF "<soft:Sample_molecule_ch1>$1</soft:Sample_molecule_ch1>\n";
}

if ($SampleRecord =~ m/!Sample_label_ch1 = (.*)/){
print RDF "<soft:Sample_label_ch1>$1</soft:Sample_label_ch1>\n";
}

if ($SampleRecord =~ m/!Sample_source_name_ch2 = (.*)/){
print RDF "<soft:Sample_source_name_ch2>$1</soft:Sample_source_name_ch2>\n";
}

if ($SampleRecord =~ m/!Sample_organism_ch2 = (.*)/){
print RDF "<soft:Sample_organism_ch2>$1</soft:Sample_organism_ch2>\n";
}

if ($SampleRecord =~ m/!Sample_characteristics_ch2 = (.*)/){
print RDF "<soft:Sample_characteristics_ch2>$1</soft:Sample_characteristics_ch2>\n";
}

if ($SampleRecord =~ m/!Sample_molecule_ch2 = (.*)/){
print RDF "<soft:Sample_molecule_ch2>$1</soft:Sample_molecule_ch2>\n";
}

if ($SampleRecord =~ m/!Sample_label_ch2 = (.*)/){
print RDF "<soft:Sample_label_ch2>$1</soft:Sample_label_ch2>\n";
}

if ($SampleRecord =~ m/!Sample_description = (.*)/){
print RDF "<soft:Sample_description>$1</soft:Sample_description>\n";
}
```

```
if ($SampleRecord =~ m/!Sample_description = Image: (.*)/){

print RDF "<soft:Sample_description>$1</soft:Sample_description>\n";

}


if ($SampleRecord =~ m/!Sample_data_processing = (.*)/){

print RDF "<soft:Sample_data_processing>$1</soft:Sample_data_processing>\n";

}


if ($SampleRecord =~ m/!Sample_platform_id = (.*)/){

print RDF "<soft:Sample_platform_id
rdf:resource=\"http://www.ncbi.nlm.nih.gov/geo/query/acc.cgi?acc=$1\"/>\n";

}


if ($SampleRecord =~ m/!sample_table_begin/){

$SampletableBegin=$cnt;

#print "SampletableBegin=$SampletableBegin\n";

}

}


@Sampletablesplice=@SampleRecord;

@Sampletable =splice(@Sampletablesplice, $SampletableBegin);

shift(@Sampletable);

pop(@Sampletable);

foreach $Sampletablerow (@Sampletable){

@Samplerowdata = split("\t", $Sampletablerow);

$S_ID = $Samplerowdata[0];

print RDF "<soft:Sample_data_table_id rdf:resource=\"$base/$sampleResource.$S_ID\"/>\n";

}

print RDF "</soft:Sample>\n";


foreach $Sampletablerow (@Sampletable){

chomp $Sampletablerow;

@Samplerowdata = split("\t", $Sampletablerow);

$VALUE= pop(@Samplerowdata);
```

```
$LOG_RAT2N_MEAN = pop(@Samplerowdata);

$S_ROWID = shift(@Samplerowdata);




print RDF "<soft:Sample_data_table rdf:about=\"$base/$sampleResource.$S_ROWID\"\>\n";

print RDF "\t <soft:sample_idref>$S_ROWID</soft:sample_idref>\n";

print RDF "\t
<soft:sample_log_rat2n_mean>$LOG_RAT2N_MEAN</soft:sample_log_rat2n_mean>\n";

print RDF "\t <soft:sample_value>$VALUE</soft:sample_value>\n";

print RDF "</soft:Sample_data_table>\n";

}




}#while

} # sub get_sample

# End of subroutine definitions
```
